# The role of brainstem biopsy and targeted therapies in pediatric diffuse midline glioma/diffuse intrinsic pontine glioma

**DOI:** 10.3389/fonc.2024.1504440

**Published:** 2024-12-23

**Authors:** Shehryar R. Sheikh, Violette M. R. Recinos, Eric M. Thompson, Ross Mangum, Mariah Wright-Nadkarni, Bradley Gampel, Neha J. Patel

**Affiliations:** ^1^ Department of Neurosurgery, Cleveland Clinic, Cleveland, OH, United States; ^2^ Department of Molecular Medicine, Case Western Reserve University, Cleveland, OH, United States; ^3^ Department of Neurosurgery, Washington University, St. Louis, MO, United States; ^4^ Center for Cancer and Blood Disorders, Phoenix Children’s Hospital, Phoenix, AZ, United States; ^5^ Pediatric Hematology/Oncology, Nationwide Children’s Hospital, Columbus, OH, United States; ^6^ Department of Pediatrics, Sylvester Comprehensive Cancer Center, Miami, FL, United States; ^7^ Department of Pediatric Hematology-Oncology and Blood & Marrow Transplant, Cleveland Clinic, Cleveland, OH, United States

**Keywords:** DMG, DIPG, diffuse midline glioma, diffuse intrinsic pontine glioma, brainstem biopsy, targeted therapy, H3K27M

## Abstract

Pediatric diffuse midline glioma (DMG), including diffuse intrinsic pontine glioma (DIPG), are aggressive brainstem tumors with a dire prognosis, traditionally diagnosed based on MRI characteristics. The recognition that molecular characteristics may determine prognosis and response to therapy has led to a reevaluation of biopsy necessity. This comprehensive review addresses the evolving role of brainstem biopsies in diagnosing and managing these tumors – both within the context of a clinical trial and in routine clinical care. We examine practice variability around brainstem biopsies for DMG/DIPG, revealing a global inconsistency in biopsy application and perceptions amongst providers. We show that safety profiles from contemporary studies demonstrate a high diagnostic success rate with minimal permanent morbidity, supporting the feasibility of biopsies in expert centers. Beyond the safety angle, we discuss the utility of biopsies in enabling personalized medicine, highlighting how molecular profiling has been used in multiple centers to guide targeted therapies. We present initial evidence from case studies and registry reports to address whether these molecularly targeted approaches are 1) clinically feasible, and 2) likely to extend survival. Furthermore, we present evidence to support the notion that biopsies facilitate the design of more refined clinical trials, shifting from a one-size-fits-all model to molecularly stratified studies. We discuss how this new paradigm for trial design is likely necessary in the context of DMG/DIPG given the lack of progress in this disease for the last several decades. Future directions discussed in the review include liquid biopsy techniques to complement or replace tissue sampling, aiming to enhance diagnostic precision and treatment monitoring.

## 1 Introduction

The role of biopsies in the diagnosis and management of children with brainstem tumors has been debated in the academic literature for several decades. The majority of primary pediatric brainstem tumors are H3K27-altered diffuse midline gliomas (DMGs), and outcomes for these patients are universally poor. The median overall survival in a contemporary registry was less than 12 months (1, 2). In a seminal report from the Children’s Cancer Group in 1993, Albright et al. argued that “MR scans provide images that are virtually diagnostic of brain stem gliomas and yield prognostic information equivalent to that obtainable from biopsies [ … ] as yet no one has demonstrated that modifications in therapy based on the biopsy results contribute to improved outcome.” (3) In the three decades since the Albright report, several investigations have called into question the notion of DMG as a radiographic diagnosis by raising concerns around inter-observer variability (4) and limited specificity (5). This has left the question of biopsy as a diagnostic necessity largely unresolved. Concurrently, a vibrant debate persists over the utility of biopsy-derived molecular information in the clinical management of DMG patients given that outcomes have historically been universally poor. It is unknown if a biopsy improves progression free survival or overall outcomes. Finally, the question of whether biopsies in the pediatric brainstem are safe in contemporary neurosurgical practice has also been a focus of the literature. Collectively, these issues constitute a complex and important debate for the pediatric neuro-oncology community with important implications for patient management.

In this comprehensive review, we aim to provide a broad overview of multiple themes related to the safety and utility of pediatric brainstem biopsies. Our analyses are focused primarily on the question of biopsies as they relate to suspected diffuse midline glioma or ‘DMG’. Consistent with the 2021 WHO CNS tumors classification scheme, we employ the term ‘DMG’ to refer to diffuse midline glioma H3K27-altered. The term *diffuse intrinsic pontine glioma* or ‘DIPG’ is of historical significance and, as of the 2016 WHO classification, refers to a subset of diffuse midline gliomas. Classically, DIPG refers to an aggressive, expansile tumor centered specifically within the pons. In this review, we employ the updated ‘DMG’ terminology except where the source literature explicitly refers to DIPG.

**Figure 1:**
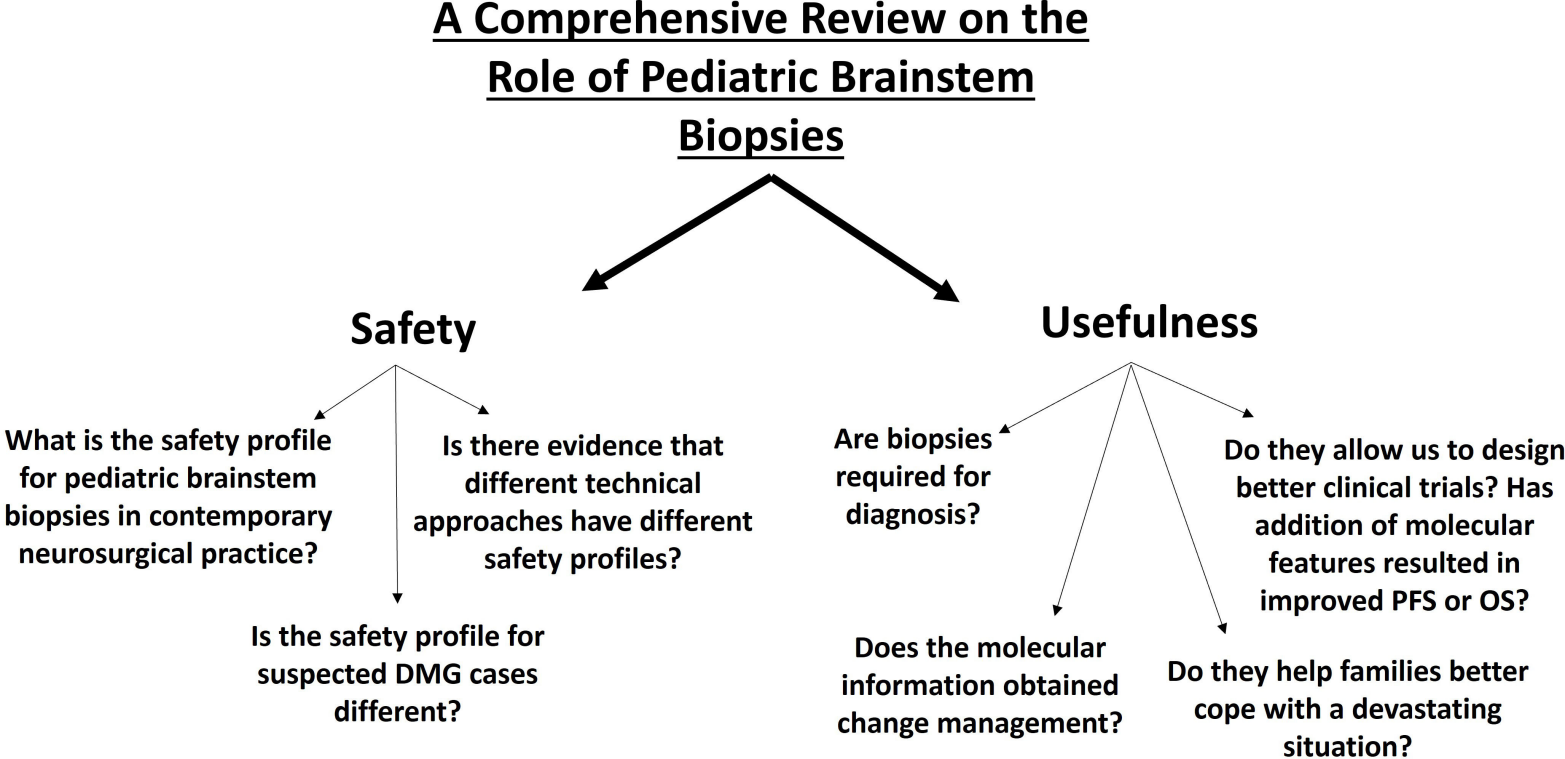
Principal issues related to pediatric brainstem biopsies in the context of DMG/DIPG.

The text is arranged in the following sections (**Figure 1**):

1. Establishing practice variability related to pediatric brainstem biopsies.
2. Safety of pediatric brainstem biopsies.
3. Utility of pediatric brainstem biopsies.

## 2 Establishing practice variability related to pediatric brainstem biopsies

The 1993 Children’s Cancer Group serves as an important historical marker in the evolution of neurosurgical practice related to pediatric brainstem biopsies (3). Published at a time when MRI was rapidly becoming ubiquitous standard of care in neuro-oncology, the report is widely cited as the basis for a shift away from using biopsies to diagnose suspected brainstem gliomas. In 1996, Fredstein and Constantini formalized MRI-based radiographic hallmarks of diffuse intrinsic pontine gliomas: diffuse pontine expansion without demarcated borders, T1 hypo-intensity with T2 hyperintensity, absent (or minimal) contrast enhancement (6). In patients with these imaging findings and a rapidly progressive clinical syndrome, they argued that “performing a biopsy, whether open or stereotactic, exposes the patient to additional unnecessary risk”. As recently as 2009, Fisher, Carson et al. argued that in patients with diffusely infiltrative brainstem gliomas, expeditious enrollment in trials with aggressive therapy was reasonable and “biopsy is rarely indicated” (7).

A marked shift in the tone of the literature related to brainstem biopsies has occurred in the last decade following the publication of multiple landmark studies that have shed new light onto the molecular underpinning of brainstem gliomas. In 2012, a series of seminal studies showed that alteration in histone H3 (most notably, lysine to methionine substitution at position 27 of H3 i.e. H3K27M) was a hallmark of the disease occurring in 70-80% of DIPGs (8–10). We now understand that H3K27M induces defective spread of the Polycomb Repressive Complex2 (PRC2) across chromatin which normally catalyzes histone methylation thereby causing gene repression (11–13). H3K27M mutation thus leads to a reduction of epigenetic gene repression driving tumorigenesis. This growing molecular understanding was reflected in the 2016 WHO CNS tumor classification scheme with the definition of the new entity “diffuse midline glioma H3 K27M-mutant” and a recognition that tumors bearing this molecular alteration are Grade 4 irrespective of histological features (14). Other less frequent but significant molecular features have been identified and have potential as druggable targets including ACVR1 (15, 16), PDGFRA (17), FGFR1, PP2A (18), EGFR (19), as well as a targetable surface antigen B7-H3 (20). It is now also appreciated that enrichment of MAPK pathway alterations correlates with long term survival in patients with H3K27M-mutant tumors (21). As these molecular targets have emerged, a palpable shift in the literature around brainstem biopsies has followed with multiple voices advocating for a more widespread use of biopsies (22–25). At a minimum, it has become accepted standard of care to discuss the risks/benefits of a biopsy within a nuanced discussion with patients and their families.

Neurosurgeons have been the most widely surveyed regarding opinions and practice patterns related to brainstem biopsies in children. In 2011, Hankinson et al. published results from a survey of 86 neurosurgeons in North America. Respondents were presented 16 representative cases (including imaging and clinical scenario) and asked to comment on whether they believed the lesion to be ‘typical’ or ‘atypical’, whether they would biopsy the lesion, and which surgical approach they would use. The study showed that no tumor was universally judged to be either typical or atypical by all neurosurgeons. In fact, 75% agreement regarding whether a tumor was typical or atypical was found in a minority (44%) of cases. Even where surgeons judged that a lesion was typical, many (range 1.2% to 66.7% based on the lesion) would still offer biopsy. When asked if, as a general matter, surgeons would offer biopsy for a typical diffuse pontine glioma as part of a multicenter trial, 69.4% said they would (4). In 2020, Tejada et al. published results from a survey of 73 neurosurgeons undertaken by the Neurosurgery Working Group of the International Society of Pediatric Oncology (SIOP) Europe Brain Tumor Group. Only 14% of surgeons felt that biopsy was necessary for the diagnosis of DIPG. 57% stated that they would only perform a biopsy within the context of a prospective trial. Notably, 65% felt that the biopsy was justified if the trial investigated molecular targets *without* planning to use its outcome directly towards treatment. In another European survey-based study from the SIOP-BTG, Khouly et al. reported responses from 74 healthcare providers of whom the majority (88%) were pediatric oncologists. 13.5% of respondents stated that they biopsied all DIPG patients, while 28.4% stated that they biopsied most. Notably, 16% said that they never biopsied DIPG patients (26).

The role of biopsy in the care of children with diffuse midline glioma continues to evolve within the neuro-oncology community. It is clear that concurrent with the growing understanding of the molecular pathophysiology of the disease and resultant molecular heterogeneity, there has been a gradual shift away from the position of treating DIPG as a purely radiographic diagnosis.

## 3 Safety of pediatric brainstem biopsies

### 3.1 What are the rates of temporary and permanent morbidity in contemporary case series particularly for suspected DMG cases?

Given the highly eloquent nature of the brainstem structures that usually form the epicenter of diffuse midline gliomas, there has been sustained interest in ascertaining accurate safety profile data related to biopsies. Several case series from around the world have been published over the last three decades. Data from these case series have been aggregated for review in a handful of meta-analyses (**Table 1**).

**Table 1:** Summary of meta-analyses on the safety profile of brainstem biopsy in children.

Meta- Analysis Study	Details of component studies	Key Findings	Comments
Kickingereder 2013 (27)	Included both adult and children, mean age 29 years. N = 1480 total cases. All stereotactic, 68% transfrontal approach.	96.2% diagnostic success, 7.8% overall morbidity (95% CI 5.6-10.2), 1.7% permanent morbidity (95% CI 0.9-2.7), 0.9% mortality (95% CI 0.5-1.4). Biopsy trajectory had no influence on outcome measures.	Widely cited due to large pooled sample size but with the significant limitation that adults and children are combined for analysis.
Hamisch 2017 (28)	N = 735 children, mean age 8 years, all stereotactic, 60% transfrontal. Included studies from 1993-2015.	96.1 diagnostic success, 6.7% overall morbidity (95% CI 4.2-9.6), 0.6% permanent morbidity (95% CI 0.2-1.4), 0.6% mortality (95% CI 0.2-1.3). Biopsy trajectory had no influence on outcome measures.	Large pediatric-only cohort, about a third of studies are from 1980s-1990s so unclear if they reflect contemporary practice. No clear description of what the specific morbidities encountered in component studies and aggregated in the meta-analysis were.
Fu 2023 (29)	N = 381 cases, all patients <21 years of age, only included patients with suspected diffuse midline glioma.	For biopsies in general: 11% overall morbidity (95% CI 4.8-18.9), 0.3% permanent morbidity (95% CI 0-2.2), 3 mortalities in the total cohort (0.8%). For stereotactic biopsies: 5.3% overall morbidity (95 CI: 1.5 – 10.7), 0% permanent morbidity (95% CI 0-1.1), no deaths	Highly selective inclusion criteria resulting in a focused analysis for pediatric suspected DMG cases, though with smaller pooled sample size.
Lu 2023 (30)	N = 99 cases, all children with mean age 9 years, all patients had stereotactic, frameless, robot-assisted biopsy	Transient complications: 10% (95% CI: 4-19%), permanent complications 0% (95% CI 0-2%), no deaths	This study describes a pediatric-only population undergoing brainstem biopsy with a cutting-edge technique (frameless, robotic stereotaxy).
Sheikh 2024 (31)	N=1002 children, 87% stereotactic.	Transient complications: 6% (95% CI 4-8), permanent complications 1% (95% CI 0.5-2). 5 mortalities in total.	Largest pooled cohort of pediatric cases. Included only contemporary series (2003-2023). Most common transient neurological complications were: 1) cranial nerve dysfunction, 2) worsening or new ataxia, 3) limb weakness. Most common permanent complications were: 1) cranial nerve dysfunction, 2) limb weakness.

A key limitation for these analyses is that data are principally derived from retrospective case-series, which limits the quality of pooled evidence. Furthermore, surgical case series with high complication rates are less likely to appear in the academic literature than studies with favorable complication rates. These limitations notwithstanding, some basic risk estimates are ascertainable:

- Across meta-analyses, the rate of permanent complications after pediatric brainstem biopsies is low: the upper limit of the 95% confidence interval (CI) of the pooled estimates does not exceed 2.2% for any of the pediatric meta-analyses. The most commonly encountered permanent complication is new or worsening cranial neuropathy or limb weakness.
- Though permanent complications are rare, temporary complications occur in a significant proportion of patients, with two of the pediatric meta-analyses reporting upper limit of 95% CI transient complication rate around 19%. The most frequently encountered are new/worsening cranial neuropathies, ataxia, or limb weakness. Studies have not consistently reported the time to resolution for these temporary complications.
- Mortalities are rare. In the largest pediatric meta-analysis (pooled cohort of 1002 children), only 5 mortalities were found (**Table 2**):

**Table 2:** List of reported mortalities from published pediatric brainstem biopsy surgical case series.

	Publication	Age and Gender	Details of mortality
1	Cheng 2020 (32)	8 M	stereotactic biopsy of pontine anaplastic astrocytoma, progressive cerebral swelling around surgical bed leading to death on post-operative day 18
2	Cheng 2020 (32)	17 F	stereotactic biopsy of pontine anaplastic astrocytoma, cerebral herniation on post-operative day 3
3	Wang 2022 (33)	9 F	open microsurgical biopsy of pontine DMG, cerebellar swelling noted intraoperatively requiring resection of part of cerebellum, death on post-operative day 2 with due to cardiorespiratory arrest
4	Wang 2022 (33)	unclear	open microsurgical biopsy, worsening cerebellar and tumor edema causing increased hydrocephalus, concurrent diabetes insipidus and electrolyte abnormalities, death on post-operative day 5
5	Wang 2022 (33)	unclear	CNS infection following open microsurgical biopsy, death on post-operative day 23

In addition to the above listed meta-analyses, one important contemporary prospective multi-center study has added notable insights. In 2018, Gupta et al. published the results of the DIPG Biology and Treatment Study (DIPG-BATS) (34). This prospective trial involved upfront biopsy of suspected DIPG prior to definitive treatment. 50 pediatric brainstem biopsies were performed at 23 institutions across the United States, with a standardized operative protocol (stereotactic, trans-cerebellar via the middle cerebellar peduncle, an initial 3mmx1mm specimen was taken for diagnosis and up to 6 total specimens could be taken). No deaths were reported related to the surgery, however 1 death occurred 2 weeks postoperatively and was attributed to disease progression. In that one patient, worsening hydrocephalus after biopsy prompted ventriculoperitoneal shunt placement. The patient continued to deteriorate with diffuse brainstem edema and punctate hemorrhage. Only one patient experienced a permanent neurological complication attributed to surgery (hemiparesis). One patient developed dysarthria that did not resolve though this was thought to be partially related to radiation. The DIPG-BATS study is valuable because it is multi-centered and highly specific to the case of pediatric patients with suspected DIPG. The major lesson of the DIPG-BATS experience is that safe stereotactic trans-cerebellar biopsies in these children are feasible in high-volume academic centers in North America.

### 3.2 What technical variations exist and is there evidence of differential safety outcomes?

Multiple surgical approaches to biopsy of brainstem lesions have been described:

1. Open microsurgical approaches were the earliest described. A suboccipital craniectomy can be used to access the floor of the fourth ventricle where sampling can be performed. Alternatively, a small retrosigmoid craniotomy can be used to sample the ventrolateral pons and cerebellopontine angle (35). In our experience, open microsurgical biopsies of brainstem lesions have become relatively uncommon in contemporary practice though they may still be appropriate in cases where it is possible to directly sample the exophytic component of atypical brainstem lesions.
2. Stereotactic approaches have been described with multiple variations (36). Frame-based and frameless stereotaxy are both commonplace. The use of robotic stereotaxy (with or without frames) has been safely used for brainstem biopsies (30). Both trans-frontal and trans-cerebellar approaches have been used, though the trans-cerebellar is now more common in our experience (37, 38). In a meta-analysis of 1002 pediatric brainstem biopsies from 2003-2023 (31), we found that 87% of cases reported were stereotactic biopsies. In the earlier half of studies (2003-2013), 90% of stereotactic cases were frame based, while in the later half (2014-2023) only 37% were frame based with the remainder being either frameless or robotic.
3. Endoscopic approaches have been described which allow for concurrent treatment of obstructive hydrocephalus (39, 40).

There have not been any trials to assess differential safety outcomes with technical variations of pediatric brainstem biopsies for presumed DMG. In their analysis of 381 cases, Fu et al. conducted a separate subgroup analysis for stereotactic biopsies finding that overall morbidity rate was 5.3% (95% CI 1.5-10.7), likely lower than the rate for the combined stereotactic and open biopsy cases (11% overall morbidity, 95% CI 4.8-18.9). There was no sub-group analysis comparing trans-frontal and trans-cerebellar approaches in terms of safety profiles. In the five mortalities we found in our meta-analysis of 1002 cases (**Table 2**), 2 deaths occurred after stereotactic biopsy (1 through a trans-occipital approach, and 1 through a trans-frontal approach) while 3 occurred after open biopsy. Overall, no conclusive evidence exists to suggest that one technical variation for pediatric brainstem biopsies is clearly safer than the other; permanent complications and death are rare with all technical variations described. It is reasonable for surgeons to implement the technique they are most experienced and comfortable with.

## 4 Utility of pediatric brainstem biopsies

### 4.1 Is biopsy necessary for diagnosis?

The paradigm of treating DMGs as a radiographic diagnosis was based in large part on the belief that diffusely expansile minimally enhancing lesions centered primarily in the pons could not represent any entity that would be surgically resected and thus empiric treatment with radiation (with or without chemotherapy) was considered reasonable. It is worth examining whether existing literature supports this notion or if, instead, evidence exists that lower grade lesions (such as pilocytic astrocytomas) can mimic DMG and a presumptive radiographic diagnosis would be misguided.

A central problem with evaluating the validity of radiographic diagnosis for DMG is the issue of interrater variability which is made even more complicated by the fact that the oncopathological definition of DMG has evolved over the decades concurrent with dramatic progress in the quality of magnetic resonance imaging. It is thus not clear whether a group of neuro-oncologists reviewing an MRI study obtained in 2024 would arrive at the same radiographic diagnosis as an equally expert group of reviewers two decades prior. Notwithstanding this problem, the issue of interrater variability is mitigated in part by the use of multi-reviewer panels as is common in well-designed prospective trials. In the DIPG-BATS study (34), patients with MRI consistent with ‘classic DIPG’ diagnosed at a large-volume academic center were considered and atypical features (if present) were reviewed by study chairs before participants could be enrolled in a trial. Notably, of the 50 children who underwent biopsy after this careful selection process, 100% received a histopathological diagnosis of DIPG. The largest case series from a single center where all patients were already diagnosed with classic DIPG on the basis of MRI findings at the time of biopsy was published by Puget et al. in 2015 (n = 130 children) (41). 100% of these biopsies resulted in a histopathological diagnosis of DIPG.

Nonetheless, there are several case series of pediatric brainstem biopsies in which non-high-grade glioma pathologies have been reported; notable examples are detailed in **Table 3**. These case series highlight the now well understood principle that non-high-grade glioma pathologies are not uncommon in the pediatric brainstem, even if they represent a minority of the total neoplastic disease in this region. A key limitation of these case series is inconsistency in the description for *why* a biopsy was being conducted with only a minority stating that a presumptive diagnosis of DMG had been made before the procedure was performed. Thus, it is difficult to ascertain whether the non-glioma pathologies identified in these case series represent true radiographic mimics of DMG (in which case, the existence of these entities would support the notion that biopsy is required for diagnosis).

**Table 3:** Summary of non-high-grade glioma pathologies reported in surgical case series of pediatric brainstem biopsy.

Study	Details of included patients and reasons for conducting biopsy (if provided)	Histological subtypes identified *(emphasis on non-glioma findings)*
Roujeau 2007 (42)	24 children all with ‘diffuse pontine lesions’	22 children had WHO III/IV gliomas. 1 child had pilocytic astrocytoma, and 1 had WHO II astrocytoma. In these low-grade lesions, up-front radiation was not used and these children were not enrolled in a trial investigating a chemotherapeutic agent for malignant tumors.
Pirotte 2007 (43)	20 children with ‘intrinsic brainstem tumors’. Biopsies were being conducted because of atypical features (either lateralization within the brainstem or strong contrast enhancement)	2 patients had pilocytic astrocytomas (1 lesion was subependymal in the pons, the other was lateralized within the pons), 2 lateralized pontine lesions were called ‘PNET’s
Schumacher 2007 (5)	142 cases of pediatric ‘brainstem disease’, analysis conducted to find correlation between radiographic diagnosis from MRI (3 radiologists) and final histopathological diagnosis	The radiologists correctly identified non-tumor disease 50-75% of the time. They correctly identified gliomas 85-91% of the time.
Patel 2009 (44)	24 stereotactic brainstem biopsies	8 patients had pilocytic astrocytomas, 2 had tuberculous lesions, 1 had an epidermoid cyst, and 1 had infarction. No details of radiographic features of these lesions were provided.
Haegelen 2010 (45)	6 biopsies in children with brainstem lesions	1 patient had gliosis (imaging characteristics not provided)
Dellaretti 2011 (46)	44 children with intrinsic brainstem lesions	Of the patients with diffuse lesions (29), non-glioma pathologies were found in 2 patients: 1 ependymoma and 1 ganglioglioma
Ogiwara 2013 (47)	7 biopsies for ‘intrinsic brainstem lesions’ in children, reason for biopsy was “atypical” feature (extension beyond the boundary of the pons, or well-margined localized enhancing portion).	1 patient had PNET, 1 patient had pilocytic astrocytoma
Manoj 2014 (48)	41 children underwent stereotactic biopsy for brainstem lesions	3 cases of pilocytic astrocytoma, 4 tuberculomas, 1 “other inflammatory”. Authors note “all the biopsies in diffuse non-enhancing lesions were gliomas”
Quick-Weller 2017 (49)	5 children with intrinsic brainstem lesions suspected to be high grade gliomas	1 child had astrogliosis, T-cell infiltration and mineralization without tumor cells, this child was observed clinically with no progression. A second child was found to have pilocytic astrocytoma. A ‘wait and see’ strategy was adopted and the lesion showed remission on radiographic follow-up.
Dawes 2018 (50)	11 robotic brainstem biopsies in children	1 patient had an inflammatory lesion (imaging characteristics not provided)
Gupta 2020 (51)	22 robotic brainstem biopsies in children	2 had pilocytic astrocytomas (imaging characteristics not provided)
Peruzzi 2024 (52)	Case report of a 19 year old female with MRI consistent with DIPG	Biopsy showed lesional cells were negative for H3K27M alterations, sequencing revealed frameshift mutation in NF1 thought to be likely driver mutation

In summary, for cases evaluated at high-volume centers with clinical course and radiographic information closely reviewed by experts, the radiographic diagnosis of DMG appears to be highly reliable. Case reports of situations where non-glioma pathologies were found in suspected DMG cases are present in the literature, but it is unclear whether the biopsies being discussed in those reports were being conducted in part because the diagnosis of DMG was uncertain. Uncontroversially, where the diagnosis of DMG is not clear based on clinical course or atypical radiographic findings, biopsy is indicated.

### 4.2 Are medical treatment schema based on molecular targets useful outside the context of trial enrollment?

The INFORM registry has provided compelling insights into the potential utility of molecular markers as the basis of targeted treatment schema for DMG patients (53). In a report from 2019, Pfaff et al. reported initial experience with 21 biopsy-proven DIPG cases submitted to the registry. They showed that it was possible to conduct molecular analysis on samples for all cases. It took 3-4 weeks (median 22 days) from arrival of tumor tissue to availability of preliminary target analysis report. This short turn-around time is vital given the aggressive clinical course of the disease.

‘Potential targetable alterations’ were identified in 16 cases. 5 of these alterations were used as the basis for targeted therapy as detailed below. Notably, patients were not enrolled in an early phase clinical trial as no matching open phase I/II trials were available in Germany at the time.

- 2 patients had genetic alteration in platelet derived growth factor receptor alpha *(PDGFRA)*, which is a cell surface tyrosine kinase receptor. These patients were treated with receptor tyrosine kinase inhibitors dasatinib or sorafenib in addition to the standard of care at their center i.e. temozolomide (TMZ) and irradiation.
- One patient had a genetic alteration in *PIK3CA* which encodes a subunit of the PI3K enzyme involved in the PI3K/AKT/mTOR pathway. This patient was treated with mTOR inhibitor everloimus in addition to radiation + TMZ.
- One patient had a *KRAS* amplification. This gene encodes a protein involved in the RAS/MAPK signaling pathway. This patient was treated with radiation as well as an inhibitor of MEK (a key enzyme in the RAS/MAPK pathway) in the form of trametinib, and cetuximab (a monoclonal antibody targeting epidermal growth factor receptor which disrupts downstream signaling pathways including RAS/MAPK).
- One patient had both a PDGFRA amplification as well as a MET fusion (also a tyrosine receptor kinase). The patient was treated with radiation and 2 tyrosine kinase inhibitors: crizotinib and dasatinib.

The INFORM registry experience with DIPG patients is most valuable as a demonstration of the real-world feasibility of efficient molecular profiling of tumors, while laying the foundations for rational molecularly targeted treatment even outside the context of a clinical trial.

In 2022, Del Baldo et al. published their experience of implementing a targeted therapies approach to 25 DIPG patients at a single academic center in Italy (Bambino Gesu Children’s Hospital, Rome). 23 patients presented at time of diagnosis and two presented at time of disease progression (having previously been treated with temozolomide and radiotherapy). All patients underwent biopsy with molecular profiling. Newly diagnosed patients were all treated with the same standard of care regimen (radiotherapy, nimotuzumab, and vinorelbine). At time of progression, patients could either receive a standard therapy (bevacizumab and temozolomide) or a targeted therapy if molecular targets were identified and the disease course was not too advanced.

In 15 cases (60%), targetable alterations were found. 9 of these patients were treated with targeted therapies at time of progression (in addition to the standard progression protocol) based on the following schema:

- patients whose tumor expressed mTOR/p-mTOR by immunohistochemistry (IHC) were treated with everolimus.
- patients with PDGFRA alterations were given pazopanib (a tyrosine kinase inhibitor).
- those with ACVR1 mutation (associated with BMP signaling) were given palovarotene (a RARγ agonist that can inhibit BMP signaling).
- one child had BRAFv600E mutation as well as mTOR/p-mTOR expression and was treated with both everolimus and vemurafenib (an inhibitor of BRAF V600E kinase).

2 year overall survival was 19.8% for the whole study population. Notably, median overall survival was longer in the targeted treatment group (20.26 months) compared to the non-targeted treatment group (14.18 months). Thus, this report provides a compelling real-world example of how molecular profiles can be used to create targeted treatment schema with preliminary evidence that some survival advantage may be accessible through this paradigm.

The INFORM registry study and the Bambino Gesu series both used existing knowledge of molecular oncopathogenic processes to create rational targeted-treatment schema. In contrast, it is possible to use biopsy-derived tumor cell lines from a patient to create *in vitro* drug response assays which can in turn be used to choose medical treatment (54). In 2019, Tsoli et al. demonstrated that cell lines could be generated for 32 out of 38 DIPG biopsy samples (84.2%) (55), though the time frame in which this was accomplished is unclear. Mueller et al. have recently published an elegant demonstration of this approach in a real-world clinical workflow (56). In 24 patients with biopsy confirmed DMG, they were able to develop patient-derived cell lines that could be used for real-time drug screening for 9 patients (38%). Median time to develop the cell line (from time of biopsy) was 2.5 months, with a range of 1.5 to 5 months. The cell lines were used to test drug response to four agents in different combinations: ONC201, ONC206, paxalisib, and panobinostat. In one case, a patient was treated with paxalisib based on the demonstrated *in vitro* drug sensitivity from the patient derived cell line.

The reports reviewed in this section present an evolving model for creating targeted treatment schema for DMG patients on the basis of biopsy derived clinical targets even outside the context of a clinical trial. Multiple centers around the world have demonstrated the feasibility of rapid molecular profiling for DMG patients, which is a pre-requisite for targeted therapies. Specialized centers are also able to generate patient-derived cell lines which can be used for drug sensitivity analyses, though technical efficacy and efficiency of this process requires continued optimization. As the Bambino Gesu report highlights, there is some initial promise that the use of targeted therapies even outside a trial context may provide an overall survival benefit to selected patients; this will undoubtedly be the subject of active inquiry in the coming years.

### 4.3 Do biopsies allow for the design of better trials?

Conventional clinical trials for DMG did not rely on biopsy derived molecular features. Inherent within this kind of trial design was the obligate assumption that the disease was sufficiently homogenous (at least in terms of its response to therapy) that a signal of improved survival for a given therapy under investigation would nonetheless become apparent. Given the lack of progress in improving survival over multiple decades of investigation using this approach to trial design, it is vital to re-evaluate the paradigm. This is especially true given that we now appreciate that DMG is a molecularly heterogenous disease with different molecular subtypes associated with different survival rates and response to therapy (57).

The DIPG-BATS trial (NCT01182350, initial results published 2018) was the first large multi-center trial to require biopsy for all children and use molecular profiling as the basis of treatment arm allocation (34). All patients were treated with bevacizumab and radiotherapy but patients could also receive erlotinib based on the presence of EGFR mutation and temozolomide on the basis of MGMT promoter methylation. Perhaps the most important lesson from the DIPG-BATS experience was that this molecular-target driven workflow was feasible. All patients except one received radiation within 21 days of biopsy and there was sufficient tissue for genetic studies and treatment arm stratification in 96% of patients.

In 2019, Mueller et al. published results of PNOC003, a pilot precision medicine trial from the Pacific Pediatric Neuro-Oncology Consortium. DIPG patients underwent biopsy and samples were used for whole exome sequencing and RNA sequencing of paired tumor and normal tissues. Based on the molecular profile, up to 4 FDA approved drugs could be administered following evaluation by a specialized tumor board. For all 15 subjects in the pilot study, a treatment plan was successfully issued within 21 business days of tissue acquisition. Taken together, the DIPG-BATS and PNOC003 experience provide evidence that clinical workflows that incorporate tumor biopsy, molecular profiling, and patient-tailored treatment plans can be implemented within clinically relevant timeframes.

In 2023, Grill et al. published results from BIOMEDE (NCT02233049): a large, international, phase II platform trial comparing 3 targeted therapies in combination with radiotherapy for patients with biopsy proven DIPG (age < 25) (58). All patients underwent biopsy and central pathological review was used to confirm H3K27me3 loss and to obtain a molecular profile. All patients received 54 Gy radiation and, depending on the molecular profile, received erlotinib (for EGFR overexpression), everolimus (for mTOR activation), or dasatinib (tyrosine kinase inhibitor, no specific biomarker was needed for this drug in the trial). 233 patients were enrolled at 45 centers in 7 countries over 5 years. Overall survival was not different between different treatment arms. The BIOMEDE experience demonstrated that international multicenter trials for DIPG involving biopsy at diagnosis and biomarker-driven treatment allocation are feasible.

At the time of writing, we found 38 early phase interventional trials for children with DIPG/DMG in the active recruitment phase on *clinicaltrials.gov*. 14 of these trials (38%) require biopsy for enrollment (**Table 4**). The reason for requiring biopsy is variable across the trials:

1. Utilization of CAR T primed against a specific antigen that must first be confirmed to be expressed by the tumor.
2. Targeted small molecules which would only be expected to be efficacious if the tumor had specific gene alterations.
3. Tumor specific ex-vivo expanded immune cells which cannot be generated without tumor sample.
4. Delivery of oncolytic agents directly into the tumor bed through a biopsy tract.
5. Some trials are requiring the confirmation of H3K27M alteration for diagnosis but do not make use of any additional molecular features in treatment arm allocation.

**Table 4:** Summary of currently enrolling DMG/DIPG trials that require a biopsy for enrollment.

NCT Number	Study Title	Description	N	Start Date (Month-Yr)	Completion Date (Month-Yr)	Location
NCT04099797	C7R-GD2.CAR T Cells for Patients With GD2-expressing Brain Tumors (GAIL-B)	Patients with GD2-expressing newly diagnosed DMG will be treated with intracerebroventricular administration of G7R-GD2 CAR T cells followed by intravenous administration	34	February-20	February-24	US
NCT04196413	GD2 CAR T Cells in Diffuse Intrinsic Pontine Gliomas(DIPG) & Spinal Diffuse Midline Glioma(DMG)	Patients with H3K27M-mutant DIPG will be treated with autologous T-cells transduced with retroviral vector expressing GD2-chimeric antigen), i.e. a type of CAR-T therapy	54	June-20	July-43	US
NCT04655404	A Pilot Study of Larotrectinib for Newly-Diagnosed High-Grade Glioma With NTRK Fusion	Patients with NTRK fusion alterations are treated with larotrectinib (Tkr inhibitor)	15	April-21	December-25	US
NCT04732065	ONC206 for Treatment of Newly Diagnosed, Recurrent Diffuse Midline Gliomas, and Other Recurrent Malignant CNS Tumors	Patients with DMG H3K27 altered who have had prior radiation will receive DRD2 antagonist/ClpP agonist ONC206	256	August-21	December-27	US
NCT04749641	Neoantigen Vaccine Therapy Against H3.3-K27M Diffuse Intrinsic Pontine Glioma	Patients with the H3.3-K27M variant are treated with vaccine	30	March-21	December-24	China
NCT04837547	PEACH TRIAL- Precision Medicine and Adoptive Cellular Therapy	Patients are treated with tumor-specific ex vivo expanded autologous lymphocyte transfer	24	September-21	September-24	US
NCT04943848	rHSC-DIPGVax Plus Checkpoint Blockade for the Treatment of Newly Diagnosed DIPG and DMG	(biopsy required for DMG, not typical DIPG, to confirm histone methylation) Patients are treated with neoantigen heat shock protein vaccine and balsitilimab (anti-PD1) and/or zalifrelimab (anti CTLA4)	36	January-22	March-24	US
NCT05278208	Lutathera for Treatment of Recurrent or Progressive High-Grade CNS Tumors	Patients with biopsy proven DIPG, with expression of SST2A and uptake on DOTATE PET will receive Lutetium 177 dotate	65	November-22	November-24	US
NCT05478837	Genetically Modified Cells (KIND T Cells) for the Treatment of HLA-A*0201-Positive Patients With H3.3K27M-Mutated Glioma	Newly diagnosed patients with H3.3K27M mutant DMG and HLA-A*0201 will be treated with KIND T cells (autologous anti-H3.3K27M TCR-expressing T cells)	12	July-23	August-29	US
NCT05544526	CAR T Cells to Target GD2 for DMG	Patients with H3K27M mutant DMG will receive CAR T therapy with GD2 CAR T cells	12	August-23	December-39	UK
NCT05717699	Oncolytic Virus Ad-TD-nsIL12 for Progressive Pediatric Diffuse Intrinsic Pontine Glioma	Patients with progressive DIPG are enrolled and undergo biopsy with concurrent placement of Ommaya reservoir through the biopsy channel. After surgery, they are treated through the reservoir with Ad-TD-nsIL12 (a novel oncolytic virus) which is delivered into the tumor.	18	January-23	January-28	China
NCT05717712	Oncolytic Virus Ad-TD-nsIL12 for Primary Pediatric Diffuse Intrinsic Pontine Glioma	Patients with newly diagnosed radiographic DIPG are enrolled and undergo biopsy with concurrent placement of Ommaya reservoir through the biopsy channel. After surgery, they are treated through the reservoir with Ad-TD-nsIL12 (a novel oncolytic virus) which is delivered into the tumor.	18	January-23	January-28	China
NCT06305910	CD200AR-L and Allogeneic Tumor Lysate Vaccine Immunotherapy for Recurrent HGG and Newly Diagnosed DMG/DIPG in Children and Young Adults	Patients with H3K27M altered DIPG/DMG will be given GBM6-AD vaccine and CD200AR-L (CD200 activation receptor ligand)	24	March-24	January-27	US

As yet, all clinical trials (irrespective of whether biopsy-derived molecular alterations were targeted) have failed to identify medical treatments that increase survival for DIPG/DMG. However, as reviewed here, multiple large multicenter trials have now demonstrated the safety and feasibility of using a biopsy-driven approach for all children diagnosed with DMG/DIPG. This new paradigm of trial design for DMG/DIPG is necessary given our existing knowledge of the molecularly heterogenous nature of the disease.

### 4.4 Do biopsies help families cope with a devastating situation?

Entirely independent of the utility of biopsies as a tool to guide treatment decisions, it is important to examine the notion that biopsies may play a role in helping the family unit better cope with a devastating situation. Holistic care of the family unit is a vital component of the care of DIPG/DMG patients; if indeed biopsies can provide a sense of direction and even hope for the family, the procedure may have some value for this reason alone.

In their qualitative study of interviews with the parents of children who had died of DIPG/DMG, De Clerq et al. have provided valuable insights into the lived experience of impacted families (59). Several key findings provide a basic framework of the family mindset in this context. One of the most prominent findings that investigators discerned from interviews was the parents’ need “to have at least the feeling that they were ‘doing something.’” Importantly, this desire to feel like *something* was being done co-occurred with the knowledge that the tumor was likely fatal. In spite of this insight, the family perceived value in taking *some* action: “So actually already from the reaction before the biopsy, one could conclude that it would go the wrong way. So between doing everything or nothing … Well, you can’t tell your kid that you’re doing nothing. Because the child wants to try to see if it’s going to be okay. (father 3)” In the same vein, speaking about their treatment path, one mother shared “…it’s still right in retrospect, because otherwise I would have a bad conscience…” This latter quotation highlights the important notion that a biopsy may fit into a family’s perception of “doing everything possible” which may prove to be a point of unification and source of emotional strength for the family unit even after the demise of the patient.

## 5 Discussion

In regards to the safety of biopsies, we have examined the evidence generated by broad systematic review of existing case series (i.e. meta-analyses) as well as evidence from contemporary trials for DMG/DIPG. Our review of this literature shows that biopsies of the pediatric brainstem are highly efficacious in yielding a diagnosis (>96%) with very low rates of permanent morbidity (<2%); this trend is particularly true for stereotactic biopsies in this region though all technical variations reviewed shared comparable safety profiles. Multiple large-scale multi-instituational trials have now been conducted with biopsy required for enrollment; safety profile of biopsy in these trial settings was similar if not better than the safety profile gleaned from historical cohorts. Given the abundance of evidence reviewed on this topic, we feel that children with suspected DMG/DIPG can safely have a biopsy at high-volume centers.

The potential usefulness of biopsies can be analyzed separately both within and outside the context of clinical trials. We feel that the use of biopsies within the context of trials is largely uncontroversial given our growing understanding of the molecular heterogeneity inherent in DIPG/DMG and evidence that molecular subtypes have different expected survival and are variably responsive to different therapies. As we have reviewed here, multiple large multi-instituational trials have now been published which used molecular target information as the basis of treatment arm allocation. Though none of these trials have clearly found superior survival outcomes associated with targeted therapies, they are nonetheless an invaluable demonstration of the fesability of large-scale targeted trials for this disease. In addition to the use of molecularly targeted therapies, biopsies have allowed for investigations using immune cells primed against a patient’s tumor (e.g. CAR T and ex-vivo expanded autologous lymphocytes) as well as the development of cell lines, organoids, and xenografts, all of which have the potential to offer insight into putative therapies.

Outside the context of a clinical trial, we have reviewed how different centers around the world have developed treatment schema to incorporate targeted therapies into their treatment of DIPG/DMG patients. These early studies have shown the feasibility of this approach. For the practicing pediatric neuro-oncologist, our review of these ‘off-study’ approaches may serve as a starting point to show the fesability of developing targeted schema in response to molecular profiles while being sensitive to personal experience and local drug availabilities.

## 6 Future directions

As enumerated above, over a dozen registered trials are currently recruiting children with DIPG/DMG for biopsy-directed investigational treatments. In the coming years, the results of these targeted therapy trials will be highly informative and will shed valuable light not only on the efficiacy of individual targeted therpies but also on the utility of the paradigm of biopsy-driven treatments as a whole.

There is enormous interest in the development of non-invasive ‘liquid biopsy’ tests in the context of DIPG/DMG with the dual intent of providing molecular profiles as well as monitoring response to treatment (60, 61). ‘Liquid biopsies’ in this context would consist of blood and CSF based laboratory tests that can detect non-cellular DNA fragments from the tumor to provide molecular information that is otherwise only available through tissue sampling. In 2017, Huang et al. reported that they had used PCR to identify the *H3F3A* c.83A > T transversion (H3.3K27M) in 4 out of 5 patient samples of CSF which was concordant with the H3K27M status obtained by analysis of tumor biopsy sample (62). The work has since been replicated and optimized by multiple groups (63, 64), with Li et al. reporting in 2021that they had developed (and validated across three institutions) an assay to detect H3K27M mutation using CSF with a sensitivity of 100% using digital droplet PCR (ddPCR) (65). The key limitation of the technology, as it has been developed so far, is that the information obtained from it is limited to select mutations for which primer pairs are developed, and so the full molecular profile that can be obtained from a tissue sample is not available. This limitation would be obviated if, in the future, the technology could use tumor DNA to perform targeted sequencing panels or whole exome sequencing. Nonetheless, we note that many DMP/DIPG trials require tissue diagnosis primarily to confirm H3K27M mutation status. If indeed a liquid biopsy could provide this limited molecular information in a highly sensitive and specific manner, it is not hard to imagine that CSF-based evidence of mutation status may be deemed sufficient for enrollment (66). In that scenario, patients would have a new ‘middle ground’ between a completely biopsy-undirected and a fully biopsy-directed treatment path. In addition to its potential role in obtaining molecular profiles, we also note the potential of liquid biopsies as a way monitoring treatment response. If future iterations of the technology could provide information analagous to ‘tumor titers’ in the CSF, we would gain a vital tool that would allow early discontinuaiton of therapies that a patient is not responding to and subsequent initiation of alterantive therapies that may be more efficacious; this would represent a significant improvement over the current standard of relying on radiographic progression.

## 7 Conclusion

The existing evidence supports the notion that contemporary pediatric brainstem biopsies are likely to be safe and moelcular profiles can be obtained in clinically relevant timeframes as a standard of care. DMG/DIPG is a molecularly heterogeneous disease and a gradual shift towards molecular target oriented trials is essential to improving known dismal outcomes. Outside of the context of a trial, targeted treatment schema made on the basis of molecular profiles have been applied and are faesible to implement. Ongoing and future studies will be centered on establishing the survival benefit of targeting specific molecular targets and on developing less invasive means of obtaining molecular profiles that could obviate the need for tissue sampling.
